# Adipokines at the crossroads of obesity and mesenchymal stem cell therapy

**DOI:** 10.1038/s12276-023-00940-2

**Published:** 2023-02-07

**Authors:** Duc-Vinh Pham, Thi-Kem Nguyen, Pil-Hoon Park

**Affiliations:** 1 College of Pharmacy, Yeungnam University, Gyeongsan, Republic of Korea; 2 Research Institute of Cell Culture, Yeungnam University, Gyeongsan, Republic of Korea

**Keywords:** Stem cells, Cell biology

## Abstract

Mesenchymal stem cell (MSC) therapy is an emerging treatment strategy to counteract metabolic syndromes, including obesity and its comorbid disorders. However, its effectiveness is challenged by various factors in the obese environment that negatively impact MSC survival and function. The identification of these detrimental factors will provide opportunities to optimize MSC therapy for the treatment of obesity and its comorbidities. Dysregulated production of adipokines, a group of cytokines and hormones derived from adipose tissue, has been postulated to play a pivotal role in the development of obesity-associated complications. Intriguingly, adipokines have also been implicated in the modulation of viability, self-renewal, proliferation, and other properties of MSC. However, the involvement of adipokine imbalance in impaired MSC functionality has not been completely understood. On the other hand, treatment of obese individuals with MSC can restore the serum adipokine profile, suggesting the bidirectionality of the adipokine–MSC relationship. In this review, we aim to discuss the current knowledge on the central role of adipokines in the crosstalk between obesity and MSC dysfunction. We also summarize recent advances in the use of MSC for the treatment of obesity-associated diseases to support the hypothesis that adipokines modulate the benefits of MSC therapy in obese patients.

## Metabolic disorders: Stem cell therapy in obesity

Adjusting the activity of a group of molecules derived from body fat might improve treatment success for mesenchymal (adult) stem cell (MSC) therapy in metabolic disorders. MSC therapy shows promise for treating obesity and other metabolic conditions, however the obese microenvironment can impair MSC potency, function, and survival. To help clarify why this happens, Pil-Hoon Park and co-workers at Yeungnam University in Gyeongsan, Korea, reviewed current understanding of adipokines, a group of signaling molecules and hormones derived from body fat tissues, and their role in the interaction between obesity and MSC dysfunction. MSCs taken from obese subjects tend to show disrupted function, including slower proliferation and lower capacity for invasion. This appears to be linked to dysregulated adipokine secretion, triggering abnormal behavior in both resident and transplanted MSCs.

## Introduction

Mesenchymal stem cells (also termed mesenchymal stromal cells or MSCs) are a heterogeneous cell population isolated from various adult tissues, such as bone marrow (BM-MSCs), adipose tissue (adipose-MSCs or ADSCs), and umbilical cord (UM-MSCs)^1^. They have postulated to originate from perivascular cells (also known as pericytes) distributed throughout the body. According to The International Society for Cell & Gene Therapy (ISCT), three perquisite attributes of MSC are plastic adherence, expression of CD105, CD90, and CD73 in the absence of CD34, CD45, CD11b/CD14, CD79α/CD19, and HLA-DR, and trilineage differentiation capacity (osteoblasts, adipocytes, and chondroblasts)^2^. In addition, since the phenotype of MSC may be altered upon in vitro expansion, other molecular markers have been developed to localize/trace MSC in situ. For example, PDGFRα^+^ cells represent a subset of perivascular cells with increased clonogenic and differentiation potentials, making PDGFRα^+^ an important in vivo marker of MSC^3^. Likewise, recent studies have led to the identification of Meflin as a potential marker of mesenchymal cells with multilineage differentiation capacity^4^.

Due to their multilineage differentiation capacity along with diverse pleiotropic activities, such as anti-inflammatory, immunoregulatory, anti-apoptotic, and anti-fibrotic effects, MSC have been widely investigated for the treatment of various diseases^1^. However, the heterogeneous nature of MSC arising from isolation methods and inter-donor variability complicates their therapeutic use. For example, since the potency of MSC is determined by the microenvironment in which they reside (i.e., the stem cell niche), metabolic syndromes, such as diabetes and obesity, which alter systemic and local milieus, have robust impacts on the fate of MSC^5^.

Obesity is a complex endocrine and metabolic disorder characterized by adipocyte hypertrophy and hyperplasia, adipose tissue inflammation and remodeling, aberrant cytokine secretion, and insulin resistance. Excess adiposity has long been considered a risk factor for the development of diverse comorbidities, including but not limited to type 2 diabetes, nonalcoholic fatty liver disease, cardiovascular disorders, osteoarthritis, and osteoporosis. However, the mechanisms underlying the linkage between obesity and these complications have not been completely elucidated. Recently, increasing attention has been given to the detrimental effects of obesity on MSC. Accordingly, obese-derived MSC exhibit impaired potency compared to lean-derived MSC that make them functionally compromised^5^. The chronic inflammatory milieu has been proposed to be partly responsible for obesity-induced MSC dysfunction, since pro-inflammatory cytokines exert strong negative effects on the proliferation, activation, and regenerative capacity of MSC. Moreover, cellular events occurring in surrounding adipose tissues, such as metabolic reprogramming, oxidative stress, and hypoxia, trigger early senescence, apoptosis, and compromise the immunomodulatory activity of MSC^6^. Yet, it remains unclear what predominantly determines the fate of MSC during obesity.

Adipokines, a group of cytokines produced and secreted by adipocytes or infiltrated immune cells in adipose tissue, function as paracrine and endocrine factors that regulate energy expenditure, metabolic homeostasis, inflammatory and immune responses, cardiovascular function, and many other physiological processes. Altered adipokine signatures, which are characterized by decreased expression of anti-inflammatory adipokines (e.g., adiponectin and omentin-1) and the upregulation of pro-inflammatory adipokines (e.g., leptin, resistin, and NAMPT/Visfatin), are thought to play central roles in the pathophysiology of obesity^7^. Since obesity may also be considered as a disease of stem cells^8^, understanding the causal relationship between adipokine imbalance and stem cell dysfunction will provide better insight into the development and progression of obesity. This review highlights the multifaceted role of adipokines in the modulation of MSC fate and the effective decision of MSC therapy for individuals with obesity.

## Functional alterations in MSC during obesity

### Effects of obesity on the survival, proliferation, and self-renewal capacity of MSC

Emerging evidence suggests that the obese environment causes a reduction in the number of tissue-resident MSC, but also leads to critical changes in the morphological characteristics, proliferation, and survival of isolated MSC during in vitro expansion. Indeed, MSC isolated from donors with obesity exhibit longer doubling times than those from lean subjects, indicating a reduced proliferative ability of obese-derived MSC^9^. Moreover, obesity slows down cell cycle progression and accelerates cellular senescence in MSC obtained from the bone marrow, subcutaneous fat, and visceral fat depots of high-fat diet (HFD)-fed mice^10^. Obese MSC were also reported to be more sensitive to apoptosis than nonobese-derived MSC^9^. Moreover, although MSC derived from lean and obese individuals share similarities in fibroblast-like morphology, obese MSC are larger in size and have more cell protrusions^9^.

Further insights into the molecular events underlying the abnormalities of obese MSC suggest that the impaired proliferation and survival of obese MSC are ascribed to genomic destabilization and dysregulation of proteins controlling cell growth and senescence. An obese environment negatively regulates telomerase activity, which in turn results in telomere shortening, genomic destabilization, and cellular aging in MSC. In addition, decreased levels of tet methylcytosine dioxygenase 1 and 2 (TET1 and TET2), which play important roles in the maintenance of genomic stability through modulation of DNA methylation, were also observed in obese MSC^9^.

Moreover, an obese environment upregulates the expression of key regulators of the cell cycle, including p16, p21, and p53, which are partly responsible for the slow growth and early senescence in obese MSC^9^. One of the critical attributes of MSC is self-renewal ability and self-maintenance of undifferentiated multipotent state. During obesity, epigenetic reprogramming of pluripotent genes, such as NANOG, OCT4, SOX2, and REX1, leads to compromised undifferentiated multipotency of MSC, which further contributes to the limited expansion and shortened lifespan of obese MSC under in vitro and in vivo growth conditions^11^.

### Effects of obesity on MSC differentiation

Multipotency allows MSC to differentiate into various cell types, such as osteoblasts, chondrocytes, myoblasts, stromal cells, fibroblasts, and adipocytes. Since the differentiation capacity of MSC is determined by the local microenvironment, it is not surprising that obesity-induced biochemical and endocrinal alterations affect MSC differentiation potential. In fact, an obese environment has been consistently reported to negatively modulate the differentiation of MSC toward osteogenic and chondrogenic lineage, suggesting that obesity compromises tissue repairing and regenerative functions of MSCs^12^. Mechanistically, the reduction in osteogenic differentiation potential might be attributed to the aberrant accumulation of reactive oxygen species in the adipose tissues of patients with obesity. Indeed, expression of NOX2 was found to be inversely correlated with the levels of osteogenic markers, such as osteonectin, alkaline phosphatase, and osterix, in MSC derived from obese patients^13^. While several studies have demonstrated the negative effects of obesity on the differentiation of MSC, further investigation is required for a better understanding how obesity impairs osteogenesis and chondrogenesis of MSC.

Unlike the effect of obesity on osteogenic and chondrogenic patterns, its effect on the adipocyte differentiation potential of MSC appears to be inconsistent across studies. MSC derived from the adipose tissue of obese donors were found to be less differentiated into adipocytes than MSCs from lean individuals^14^. Likewise, diet-induced obesity triggered a significant delay in the adipogenic differentiation of adipose-derived MSC^15^. Conversely, transcriptomics analysis showed that obesity led to the upregulation of adipogenic and inflammatory genes in MSC^16^. In addition, an altered bone marrow microenvironment in HFD-fed obese mice directed differentiation of BM-MSC to adipogenesis^17^. While the reasons underlying the differential modulation of adipogenic differentiation by the obese environment remain to be clarified, understanding role of obesity in promoting the adipogenic differentiation capacity of MSC would provide a better explanation of the pathological events that occur during obesity, including adipocyte hyperplasia and increased fat infiltration of bone marrow. In addition, emerging evidence suggests that adipose tissue contains distinct subpopulations of mesenchymal progenitor cells/preadipocytes that exhibit different metabolic phenotypes and behave differentially in response to cytokines and hormones^18^, suggesting that changes in the differentiation fate of obese adipose-derived MSC may be due to differences in the distribution of mesenchymal progenitor subpopulations in obese adipose tissue compared to their lean counterparts. Additionally, the heterogeneity of adipose tissue may contribute to the opposite observations in the adipogenesis capacity of obese MSC among different studies^14,17^. Therefore, characterization of obese adipose tissue components would provide better insight into the mechanisms underlying alterations in the differentiation pattern of obese MSC.

### Effects of obesity on the immunoregulatory function of MSC

MSC exhibit potent immunosuppressive effects by modulating the proliferation and differentiation of various immune cell populations, including T cells, B cells, natural killer cells, dendritic cells, and macrophages. However, immunosuppressive activity is not an inherent property of MSC but is induced by the inflammatory microenvironment. The presence of pro-inflammatory cytokines has been postulated to be required for the activation of anti-inflammatory and immunosuppressive MSC phenotypes. In contrast, chronic low-grade inflammation in obese patients appears to impede the immunoregulatory functions of MSC. For example, obese adipose-derived MSC exhibited significant impairment in the suppression of T-cell proliferation^19^. More surprisingly, Strong et al. reported that conditioned media (CM) from obese MSC further enhanced T-cell proliferation instead of suppressing it, as observed in response to lean MSC-derived CM^20^. Furthermore, obese MSC increased the expression of pro-inflammatory cytokines, suggesting that obesity reprograms MSC toward a pro-inflammatory phenotype^21^.

Due to their immunomodulatory characteristics, MSC have been subjected to many preclinical and clinical investigations for the treatment of inflammation- and autoimmune-associated disorders, such as graft-versus-host disease (GVHD), sepsis, and inflammatory bowel disease (IBD). However, while MSC from healthy donors showed promising therapeutic potential against these disorders, limited in vivo data indicate that MSCs from subjects with obesity, especially those with comorbid metabolic syndromes, failed to elicit these effects. In a mouse model of renal artery stenosis, obese MSC neither improved renal function nor decreased renal infiltration of M1 macrophages^22^. In another study, the administration of obese MSC even exacerbated autoimmune encephalomyelitis in a mouse model of multiple sclerosis^20^. These observations imply that MSC derived from obese individuals might not be a suitable cell source for stem cell therapy.

### Effects of obesity on other functional attributes of MSC

The therapeutic potential of MSC is strengthened by their ability to migrate to injured areas. However, MSC derived from obese subjects exhibited lower migration and invasion capacities than those from lean donors^23^. Furthermore, obese MSC exhibited impaired migration in response to various chemoattractants, which resulted in reduced homing and mobilization of MSC during tissue regeneration^23^. In addition, obesity negatively affects the angiogenic activity of MSC, as evidenced by increased secretion of anti-angiogenic factor (TSP-1) and impaired formation of capillary-like structures in vascular endothelial cells by obese-derived MSC^24^. Furthermore, obese MSC were unresponsive to an angiogenic stimulus (phorbol 12-myristate 13-acetate), whereas the angiogenic potential of nonobese MSC was significantly enhanced in the presence of this stimulus^23^.

The pleiotropic effects of MSC are mostly mediated via the secretion of various soluble proteins, including cytokines, chemokines, growth factors, and many other functional proteins. Interestingly, recent studies have revealed that obesity has a significant impact on the content of MSC secretomes^25,26^. For example, MSC derived from HFD mice increased secretion of factors related to IL-1 pathway, granzyme A signaling, and methionine degradation that supports a pro-inflammatory role of obese MSC. The secretomes of obese-derived MSC also include factors that negatively modulate osteogenesis and angiogenesis, such as Ostf1 (osteoclast stimulation factor 1) and Tgm2 (transglutaminase 2). In contrast, many proteins that play essential roles in controlling cellular redox status, angiogenic activity, and bone formation, normally secreted by MSC were found to be absent from the secretomes of obese MSC^25^. These findings suggest that the compromised functions of obese MSC are partly ascribed to obesity-induced alterations in their paracrine activity.

**Fig. 1:**
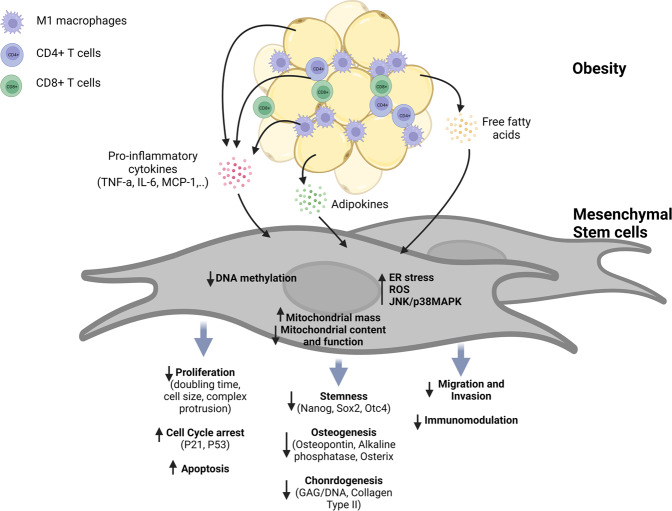
MSC reprogramming by the obesity microenvironment. The low-grade chronic inflammatory microenvironment of obesity recruits innate and adaptive immune cells, including M1 macrophages, CD4 + T cells, and CD8 + T cells, leading to the enhanced production of pro-inflammatory cytokines, such as TNF-α, IL-6, and MCP-1. Moreover, adipose-specific cytokines (i.e., adipokines) become dysregulated during obesity. In addition, the elevation in the levels of free fatty acids in the surrounding microenvironment induces lipotoxicity in MSC and eventually dysfunction of MSC. Obese microenvironment causes DNA destabilization, which in turn induces telomere shortening, cellular senescence, and apoptosis. Oxidative stress promoted by the obese microenvironment impairs mitochondrial content and function and induces ER stress and cellular ROS accumulation. These factors contribute to reduction in cell survival and proliferation, stemness, and differentiation potential into osteocytes, as well as the immunomodulatory function of obese-derived MSC.

Collectively, obesity may negatively affect the functionality of MSC through induction of early senescence program, limited proliferation, decreased differentiation and plasticity, and compromised angiogenic and immunoregulatory effects (Fig. 1). On the one hand, these alterations cause dysregulation of inflammatory and immune responses, as well as deficits in tissue repair and regeneration, which further contribute to the progression of obesity-linked metabolic complications. On the other hand, obesity would severely restrict the use of autologous MSC in the treatment of various diseases. It should be noted that the current understanding of the alterations in the phenotype and functional properties of MSC under obese conditions is mainly derived from cultured MSC, while impacts of obesity on tissue-resident MSC remain to be investigated, possibly due to technical drawbacks in characterizing the morphological and functional alterations of obese MSC in vivo. Taking into consideration the heterogeneity of MSC and that cell culturing would significantly alter the properties of MSC, the development of novel tools would be required to obtain further insights into how obese environment affects the in vivo characteristics of MSC.

## Determinants of MSC fate in the obesity microenvironment

It has become clear that obesity causes detrimental effects on the multipotency of MSC. However, the molecular mechanisms underlying obesity-induced alterations in MSC characteristics are poorly understood. In fact, while various potential underlying mechanisms have been proposed, previous findings on the functional abnormalities of obese-derived MSC have not been consistent, suggesting that MSC fate would be determined by specific factors in the obese environment, which may differ among obese patients. This section highlights the putative roles of the various factors that determine the fate of MSC in the obese state.

### Chronic low-grade inflammation

Although obesity is commonly recognized as a metabolic and endocrine disorder, it may also be considered a disease of chronic inflammation. Obese adipose tissue is characterized by the infiltration of immune cells that secrete large amounts of pro-inflammatory cytokines. Moreover, dysfunctional adipocytes have increased production of pro-inflammatory adipokines, such as leptin, NAMPT/Visfatin, resistin, and chemerin, which further promote local and systemic inflammatory responses in obese subjects^27^. As indicated earlier, inflammatory signaling is known to activate the immunosuppressive phenotype of MSC. Intriguingly, chronic inflammatory conditions have been reported to induce dysfunction of MSC. For example, BM-MSC derived from patients with rheumatoid arthritis exhibited a premature senescence phenotype, as corroborated by decreased proliferation, impaired clonogenicity, and telomerase shortening^28^. Likewise, the inflammatory environment in osteoarthritis causes MSCs to have impaired adipogenic and chondrogenic differentiation capacity^29^. Moreover, inflammation-promoted cellular aging was also observed in hematopoietic stem cells. Collectively, chronic inflammation might serve as a common negative modulator of lifespan in multipotent cells.

MSC exhibit two opposing phenotypes known as MSC1 (pro-inflammatory) and MSC2 (anti-inflammatory) in a similar manner to macrophages^30^. The transformation of naïve MSC to the MSC1/MSC2 phenotype depends on the nature and intensity of inflammatory stimuli. During the acute phase of inflammation, high levels of pro-inflammatory cytokines, such as TNF-α, IFN-γ, and IL-1β, potentiate the anti-inflammatory and immunosuppressive actions of MSC. On the contrary, long-term exposure to relatively low levels of both pro- and anti-inflammatory cytokines, which is observed in patients with obesity, drives MSC to an immune-activation phenotype. This notion is further supported by experimental evidence showing that licensing with LPS enhances the immunosuppressive potency of MSC, whereas repeated stimulation of these cells with LPS fosters cellular senescence^31^. In fact, pro-inflammatory phenotype has also been described for MSC isolated from obese individuals, in which obese-derived MSC showed increased expression of cytokines, including MCP-1, IL-6, and IL-8^21^. These cytokines originating from MSC origin, in turn, attract more immune cells to obese tissues and eventually trigger a vicious cycle of inflammation-stem cell dysfunction.

### Metabolic perturbations

An obese environment can promote metabolic rewiring in different cell types. For example, obesity causes excess accumulation of intracellular lipids, leading to impaired glycolytic capacity and immunosurveillance in natural killer cells^32^. Lipid overload may also contribute to myocardial dysfunction by decreasing the metabolic plasticity of cardiomyocytes^33^. Limited data have shown that MSC derived from obese patients with type 2 diabetes had lower glycolytic capacity than control cells^34^. Consistently, using a diet-induced obese mouse model, we observed a metabolic shift from glycolysis to oxidative phosphorylation (OXPHOS) in obese adipose-derived MSC under in vitro culture (unpublished data). Importantly, glycolysis appears to be required for multipotency and immunoregulatory activity of MSC. It has been speculated that MSC at early passages exhibit a glycolytic phenotype, and 97% of cellular ATP is generated by glycolysis compared to only 3% by OXPHOS^35^. In addition, the enhanced immunosuppressive function of TNF-α/IFNγ-primed MSC was attributed to increased glycolytic flux through upregulation of HIF-1α^36^. By contrast, mitochondrial biogenesis and oxygen consumption rate (OCR) were increased during adipogenesis of MSC, suggesting a promoting role of OXPHOS in adipocyte differentiation^37^. These findings raised a possibility that aberrances in the immunomodulatory and differentiation properties of obese-derived MSC are attributed to cellular metabolic reprogramming toward OXPHOS promoted by the obese environment.

In addition to impaired glycolytic flux, mitochondrial dysfunction was also reported in obese MSC. Accordingly, mitochondrial biomass in MSC was increased under obese conditions, accompanied by a higher OCR^9^. Due to enhanced mitochondrial respiration, obese-derived MSC produce more ROS, which are potent inducers of cellular aging. Mechanistically, lipid perturbations may be the principal drivers of mitochondrial dysfunction in obese-derived MSC. Free fatty acids, such as palmitate, may cause mitochondrial fragmentation and mitochondrial membrane potential disruption and induce apoptotic cell death, growth arrest, and early senescence in MSC^38^. Taken together, current evidence suggests a central role of chronic inflammation and dysregulated lipid metabolism in the reprogramming of MSC viability during obesity.

## Adipokines as emerging factors in the regulation of MSC fate during obesity

Adipokines are well-known as key regulators of inflammatory responses and cellular metabolism. Interestingly, emerging evidence indicates that individual adipokines modulate the plasticity, differentiation capacity, proliferation, senescence, and survival of MSC in different contexts. Herein, we provide an overview of the beneficial and detrimental effects of typical adipokines on the lifespan and function of MSC. The putative role of these adipokines in the impaired potency of obese-derived MSC will also be addressed.

### Modulation of MSC fate by leptin

Leptin, a 16-kD polypeptide, is the first adipokine to be discovered and is predominantly produced by adipocytes. Since serum leptin levels exhibit a direct correlation with body fat mass in individuals with obesity, its role in the development of obesity-linked complications has received a great deal of attention. In fact, leptin signaling is implicated in the initiation and progression of various diseases, including, but not limited to, cardiovascular disorders, type 2 diabetes mellitus, and malignancies^39^. The effects of leptin on target tissues are mediated via its membrane receptors (Ob-R), which directly couple with JAK/STAT but can also transactivate other intracellular signaling pathways, such as the PI3K/Akt, MAPK, and AMPK pathways^40^.

Although leptin is primarily secreted by adipocytes, many other cell types, including MSCs, are known to express leptin. Notably, leptin receptor has been identified as a marker of a BM-MSC subpopulation, which represents an important source of bone cells in the hematopoietic niche^41^. Therefore, it is not surprising that the roles of leptin and its receptors in the osteogenic potential of BM-MSCs are the best-characterized research area with regard to the relationship between leptin signaling and MSC function. Compelling evidence shows that leptin receptor-mediated signaling regulates the balance in osteoblast and adipocyte differentiation in adult bone marrow (Table 1). Under physiological conditions, leptin appears to inhibit adipogenesis but promotes osteogenesis in BM-MSC by activating the JAK/STAT signaling pathway^42^. Interestingly, endogenous production of leptin by MSC has been postulated to critically contribute to their osteogenic potential. This notion is supported by evidence that leptin-overexpressing BM-MSC possesses a higher osteogenic differentiation capacity than wild-type cells^43,44^. In this context, genetic engineering of BM-MSC to increase leptin expression may serve as a promising strategy to improve the therapeutic efficacy of MSC in osteoporotic patients^45^. These findings also raise the question of whether increased leptin levels during obesity will protect obese patients from osteoporosis. Although obesity was initially thought to prevent bone loss^46^, recent epidemiological and experimental evidence has revealed that obesity and osteoporosis share common genetic properties, environmental factors, and progenitor MSC^47^, which has led to the re-evaluation of the obesity-osteoporosis relationship. Unlike MSC derived from healthy individuals, osteoporotic MSC failed to respond to leptin, as evidenced by the loss of adipogenic suppression in the presence of exogenous leptin, highlighting that osteoporosis may be caused by a reduction in the responsiveness of MSC to leptin^48,49^. More surprisingly, Yue et al. recently reported that a high-fat diet (HFD) drastically elevated serum leptin levels and led to increased adipogenesis and decreased osteogenesis in BM-MSC^50^. Notably, HFD-induced bone loss was mediated by the local actions of leptin via leptin receptors on BM-MSC. Again, negative modulation of osteoblast differentiation by leptin during obesity was mediated by activation of the JAK/STAT pathway, which was also involved in the leptin-mediated promotion of osteogenesis under normal conditions^42^. Thus, the leptin/leptin receptor may play a role as a negative or positive modulator of osteoblast differentiation in a context-dependent manner. While leptin promotes osteogenesis by BM-MSC in under basal conditions, long-term exposure to high concentrations of leptin, which occurs in obesity, causes an abnormal leptin response leading to impaired osteoblast differentiation.

**Table 1:** Effects of leptin on the lifespan and functional properties of MSC.

Type	Experimental conditions	Effect of leptin on MSC	Reference
Differentiation	Healthy BM-MSC overexpressing leptin or treated with exogenous leptin	Inhibited adipogenesis but promoted osteogenesis	^45,47^
	Osteoporotic rat-derived BM-MSC stimulated with leptin	Failed to induce osteogenesis	^48,49^
	HFD obese mouse-derived BM-MSC	High serum leptin levels inhibited osteogenesis but promoted adipogenesis	^50^
Cell survival, proliferation, and senescence	BM-MSC preconditioned with hypoxia	Leptin upregulation by hypoxia conferred survival advantages to MSC by activating autophagy	^53^
	BM-MSC overexpressing leptin	Decreased apoptosis under hypoxic and serum deprivation culture conditions; increased viability upon transplantation	^55^
	UM-MSC treated with leptin or serum from systemic lupus erythematosus (SLE) patients	Long-term treatment with leptin or SLE serum containing high levels of leptin-induced cell senescence.	^56^
	ob/ob mice treated with leptin	Leptin treatment increased the yield of MSC in the adipose-derived stromal vascular fraction and enhanced the expression of MCP-1 in adipose tissue and in ob/ob mouse-derived MSC	^58^
Therapeutic effects	BM-MSC preconditioned with hypoxia	Hypoxia-induced endogenous leptin expression was required to enhance the cardioprotective effects of MSCs	^54^
	BM-MSC overexpressing leptin	Enhanced the protective efficacy against myocardial infarction	^55^

In addition to modulating adipogenic/osteogenic differentiation, leptin may also affect the fate of MSC by regulating cell survival, proliferation, and senescence. It has been suggested that short-term preconditioning with stressful conditions, such as hypoxia and serum deprivation, significantly improves the therapeutic potency of MSC, partly through enhancing cell viability upon transplantation^51^. Intriguingly, culturing MSC under serum deprivation or hypoxic conditions robustly induced leptin gene expression^52^. Moreover, favorable effects of these priming methods disappeared in leptin- or leptin receptor-deficient MSC^53,54^, suggesting that leptin signaling is required for enhanced cell survival in stressful situations. Leptin was subsequently found to potentiate the therapeutic effects of MSC by reprogramming cellular energy metabolism. In particular, leptin upregulates Optic atrophy 1 (OPA-1), a key regulator of mitochondrial fusion, thereby maintaining mitochondrial integrity and increasing glycolytic flux, which in turn prolongs the lifespan and enhances metabolic activity^55^. Although these findings generally support the role of leptin as a promising MSC priming agent, they do not necessarily indicate a beneficial effect of leptin on MSC viability and function in pathological contexts, including obesity. Indeed, while short-term leptin treatment leads to the activation of MSC, the consequence of long-term exposure to leptin, which occurs during obesity has been under-investigated. Considering this aspect, Chen et al. have reported that increased senescence of MSC isolated from patients with systemic lupus erythematosus is referred, at least in part, to high serum levels of leptin^56^. Furthermore, 3 days of treatment with leptin was sufficient to induce a senescence phenotype in control MSC by PI3K/Akt-mediated upregulation of p21 and p53 expression. Moreover, our preliminary observations also suggest that leptin triggers inflammation-associated programmed cell death in adipose-derived MSC. In another interesting discovery, expression of the leptin receptor was found to directly correlate with the number of cell divisions during the long-term culture of MSC^57^. Although the significance of leptin receptor upregulation remains unclear, the authors suggested that the leptin receptor was a marker of cellular aging.

Obesity is a multistage progressive disorder that is initially characterized by adipocyte hyperplasia followed by adipose tissue infiltration of immune cells and local inflammatory responses. In contrast to its physiological role as a negative modulator of preadipocyte proliferation and differentiation, leptin has been shown to promote adipogenesis in the MSC of HFD-fed obese mice^50^. Likewise, injection of leptin into leptin-deficient ob/ob mice resulted in a marked increase in the yield of MSC in the adipose stromal vascular fraction^58^. In this study, leptin replacement also led to increased expression and secretion of MCP-1, a potent leukocyte chemoattractant, in the adipose tissues of ob/ob mice. Additionally, leptin upregulated MCP-1 expression in adipose-derived MSC^58^. These observations support the notion that leptin may play a central role in the pathophysiology of obesity through activation of a pro-inflammatory phenotype and alterations in differentiation fate in adipose MSC. The inflammatory environment itself is known to activate MSC; however, chronic inflammation also accelerates MSC senescence^28^. More direct and convincing evidence is needed to determine the contribution of leptin to the impaired function of MSC in the later stages of obesity. Another gap in our current understanding of leptin-mediated modulation of MSC function is how leptin affects immunomodulatory potency, which is one of the most important pharmacological properties of MSC that are widely applied for the treatment of inflammation and autoimmune-associated disorders. Further insights into the effects of leptin on MSC fate will suggest new strategies to improve the therapeutic effectiveness of MSC therapy as well as restore the function of obese patient-derived MSC.

### Modulation of MSC fate by adiponectin

Adiponectin is a unique adipokine in that its serum concentration is inversely proportional to the degree of adiposity, even if it is mainly produced by adipocytes^7^. It has been considered that adiponectin exerts many favorable effects on metabolism, cardiovascular function, and the regulation of inflammatory and immune responses. Hence, impaired adiponectin signaling has been supposed to be involved in obesity-associated metabolic disorders^59^. A large amount of adiponectin is found in circulation where it may exist in various forms, including monomers, trimers, hexamers, or higher oligomers, in addition to globular adiponectin, which is cleaved and possesses only a globular domain^7^. The biological actions of adiponectin are mainly mediated via adiponectin receptor type 1 and type 2 (AdipoR1 and AdipoR2), both of which are G protein-coupled receptors^60^. Despite the high structural homology, there are considerable differences in the distribution and function of these two receptors. In particular, AdipoR1 is expressed ubiquitously and at higher levels in skeletal muscle, whereas the expression of AdipoR2 is mostly restricted to the liver. With regards to molecular actions, adiponectin stimulation of adipoR1 and adipoR2 results in activation of AMPK and PPARs, respectively, which together regulate inflammatory responses, as well as glucose and lipid metabolism. More recently, T-cadherin is also recognized as a receptor for hexameric and high-molecular-weight adiponectin that mediates the cardioprotective effects of adiponectin. Since T-cadherin does not contain an intracellular domain, it is thought to function as a coreceptor for other signaling receptors^60^.

As in the case of leptin, adiponectin signaling has also been widely associated with osteogenic/adipogenic potential of MSC. A large body of in vitro and in vivo data indicates that adiponectin plays an essential role in the maintenance of fat/bone balance by inhibiting adipogenesis but promoting osteogenesis. Indeed, treatment of MSC of different origins with adiponectin increased osteogenic differentiation^61,62^. This effect appears to be mediated via AdipoR1, since AdipoR1-deficient MSC fail to respond to osteogenic stimulation by adiponectin^62^. Mechanistically, adiponectin/AdipoR1 signaling modulates osteoblast/adipocyte differentiation through various signaling mechanisms, including activation of APPL1/AMPK, ERK and GSK-3β/β-catenin^62–64^. Moreover, adiponectin also shows beneficial effects on bone tissue regeneration by regulating the mobilization and recruitment of BM-MSC to injury sites via modulation of chemotaxis-related genes, including CXCL-1, CXCL-8, and SDF-1^65,66^. A recent study showed that ectopic expression of adiponectin might be a useful tool to enhance the effectiveness of MSC in bone regeneration^61^. Given the modulatory role of adiponectin in adipogenesis and osteogenesis of MSC, deficits in adiponectin signaling, as observed in obesity and related metabolic disorders, may contribute to aberrant adipocyte proliferation and decrease the efficacy of MSC therapy for bone loss.

**Fig. 2:**
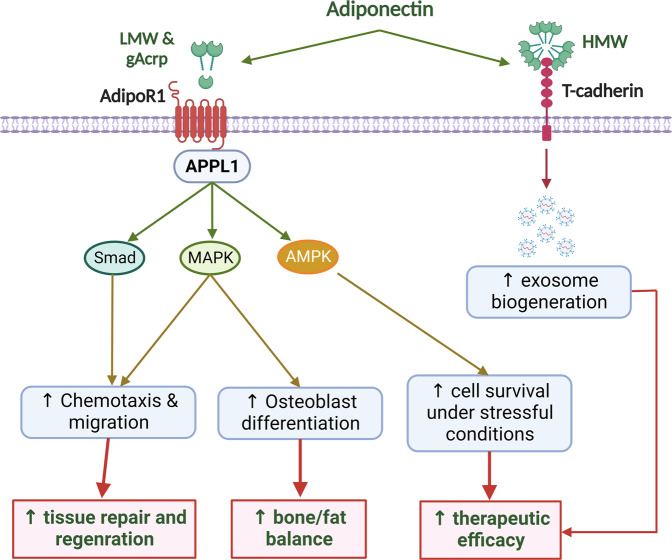
Effects of adiponectin on physiological functions and therapeutic efficacy of MSC. Adiponectin exists in different isoforms, including a cleaved form (globular adiponectin), monomers, low molecular weight (LMW), and high-molecular weight (HMW) oligomers, which can bind to transmembrane receptors, such as AdipoR1, AdipoR2, and T-cadherin. In MSC, adiponectin/AdipoR1 signaling plays important roles in the maintenance of stem cell survival and potency. In particular, adiponectin activates several cellular signaling pathways, including AMPK, MAPK, and Smad pathways, to stimulate osteoblast differentiation, chemotactic activity, and migration capacity, which together support bone repairing and regenerative function of MSC. AMPK signaling is also required for the improved survival of MSC under stressful conditions, such as hypoxia and nutrient depletion. Furthermore, T-cadherin-mediated signaling induces the biogenesis and release of exosomes, which mediate the therapeutic effects of MSC.

Some lines of evidence also indicate that adiponectin confers survival and functional advantages to MSC. One of the biggest challenges in MSC therapy is a low rate of cell survival and engraftment upon transplantation due to unfavorable conditions at the transplantation site, such as hypoxia and nutrient depletion^67^. Interestingly, adiponectin can protect MSC from apoptosis induced by serum deprivation and hypoxia^68^. Likewise, adiponectin enhances the viability of MSC under flow shear stress that simulates a stressful condition encountered during in vivo transplantation^69^. Current evidence also suggests that activation of AMPK, a key regulator of cellular responses to stress, critically contributes to the modulation of MSC fate by this adipokine^68,69^. Furthermore, the cardioprotective properties of MSC upon intravenous administration were demonstrated to be dependent on circulating levels of adiponectin^70^, further supporting the critical role of adiponectin signaling in determining the therapeutic efficacy of MSC. To increase the therapeutic potential of MSC, adiponectin stimulates the biogenesis and release of exosomes through T-cadherin-mediated signaling. Notably, the improvements in cardiac function in response to MSC injection were clearly attenuated in adiponectin-knockout mice, whereas elevating serum adiponectin levels by pretreatment with a PPAR-γ agonist further enhanced the effectiveness of MSC therapy^70^. In addition, modification of MSC using an adiponectin-expressing vector demonstrated benefits under certain conditions^71^. Based on these interesting findings, it may be predicted that the recipients with low levels of adiponectin will receive fewer benefits from MSC therapy. If this is the case, obese patients will need to be more carefully monitored for the efficacy of MSC therapy, and restoration of adiponectin signaling may be helpful to such recipients. In summary, adiponectin positively influences the survival and function of MSC in various ways. The modulatory effects of adiponectin on the physiological functions and therapeutic efficacy of MSC are illustrated in Fig. 2.

While accumulating evidence reveals the beneficial effects of adiponectin on the function and therapeutic efficacy of MSC, it remains unclear whether impaired adiponectin levels and/or adiponectin resistance are involved in the dysfunction of obese-derived MSC. Also, how obese-derived MSC responds to the restoration of adiponectin signaling will be another interesting question. Fulfilling these knowledge gaps will provide a better understanding on the molecular mechanisms underlying the decreased potency of obese-derived MSC and suggest promising strategies to improve the effectiveness of autologous MSC therapy in obese patients.

### Modulation of MSC fate by apelin

Apelin is an endogenous peptide that exists in many isoforms with different molecular sizes ranging from 12 to 36 amino acids^72^. Although it has been considered an adipokine, apelin and its receptor (APJ) are ubiquitously expressed in a variety of tissues, including brain and peripheral tissues. Apelin/APJ signaling has been implicated in the regulation of cardiovascular function, energy metabolism, angiogenesis, and fluid homeostasis^72^. It is interesting to note that while apelin exerts protective effects against various pathological disorders, including cardiovascular and metabolic diseases, its expression is often found to increase in these conditions^72,73^. With regard to obesity, some studies have shown that circulating apelin levels are higher in individuals with obesity, especially those with diabetic comorbidities^74^. Nevertheless, reduced serum apelin levels were also reported in patients with obesity and diabetes compared to the subjects with non-diabetic obesity and in obese children than in nonobese children^75^. These contrasting results suggest that other factors, such as insulin levels, rather than adiposity itself, determine the circulating levels of apelin in obesity.

The regulatory effects of apelin on the survival, proliferation, differentiation capacity, and therapeutic potency of MSC have been widely investigated. Having been known as a mitogenic factor in various cell types, apelin has also been demonstrated to stimulate the proliferation of MSC in hypoxic culture conditions^76^. Moreover, this adipokine protected MSC from apoptosis induced by various stressful stimuli, including serum deprivation, hypoxia, and oxidative stress^77–81^. The beneficial effects of hypoxic preconditioning on MSC survival were also thought to be involved in the activation of the apelin/APJ pathway^76^. Importantly, increased cell survival is accompanied by improved functionality of MSC, as evidenced by enhanced cardiogenic differentiation, osteogenesis, and angiogenic activity^78,80,82^. Furthermore, in vivo therapeutic potential of MSC transplantation against osteoporosis, myocardial injury, and hindlimb ischemia was strengthened by either apelin pretreatment or concomitant administration of MSC and apelin. In addition to supporting cell proliferation and survival, apelin has been shown to rejuvenate aged MSC and increase the cardioprotective effect of MSC in a mouse model of myocardial infarction^83^.

With regard to the mechanisms underlying modulation of MSC fate by apelin, autophagy induction was supposed to play a critical role in the enhancement of cell viability and therapeutic potency. In fact, autophagy, which is a degradative and recycling mechanism for intracellular organelles, has been suggested to be essential for protecting MSC against stressful conditions in in vitro culture as well as at transplantation sites^84^. Meanwhile, apelin induces autophagy in MSC through the activation of AMPK signaling pathway^81,83^. Alternatively, apelin activates mitophagy, a type of autophagy for the selective degradation of dysfunctional mitochondria, to ameliorate cellular oxidative stress in BM-MSC^80^. The regulation of autophagic flux was indispensable for MSC survival and proliferation during hypoxia, protection against senescence, and maintenance of osteogenic function in the presence of oxidative stress^76,80,83^. In addition to autophagy, ERK and PI3K/AKT activation were required for enhanced MSC viability. In particular, activation of AKT signaling contributed to apelin-stimulated proliferation of MSC by upregulating GSK-3β/cyclin D1 signaling^77,85^. Collectively, current evidence suggests that targeting apelin/APJ signaling may serve as a promising strategy for improving the efficacy of MSC therapy. However, due to the complexity in the relationship between the pathophysiology of obesity and apelin signaling, the role of apelin in MSC dysfunction during obesity remains to be further investigated.

### Modulation of MSC fate by other adipokines

Other adipokines, such as resistin, NAMPT/Visfatin, and omentin-1, have also been shown to play a certain role in the modulation of MSC fate, although current evidence is quite limited. Resistin and NAMPT/Visfatin are pro-inflammatory adipokines that are upregulated during obesity in a similar manner to leptin^7^. In contrast, excess adiposity results in the downregulation of omentin-1, an adipokine possessing favorable effects on the regulation of inflammation, metabolism, and cardiovascular function^40^. As in the case of many other pro-inflammatory cytokines, transient exposure to resistin led to enhanced activation of MSC. Preconditioning with resistin improved the therapeutic effectiveness of MSC against myocardial injury, partly through promoting proliferation, migration, and cardiac homing of MSC^86^. Conversely, long-term treatment of MSC with resistin has been found to reduce proliferation rate and the levels of stemness markers, and induce insulin resistance, which is a common consequence of adipose tissue remodeling in obesity^87^. Likewise, NAMPT/Visfatin promoted an inflammatory phenotype and changes in matrix-degrading enzyme profile in BM-MSC during osteogenesis, suggesting a role of NAMPT/Visfatin in impaired bone remodeling^88^. Furthermore, NAMPT/Visfatin altered the phenotypic properties of adipose-derived MSC and thereby promoted the malignant behaviors of breast cancer cells^89^. These findings support the hypothesis that increased expression of pro-inflammatory adipokines, other than leptin, such as NAMPT/Visfatin and resistin, contributes to the altered functions of MSC in obese individuals. In contrast to NAMPT/Visfatin and resistin, omentin-1 exhibited beneficial effects on the proliferation, survival, and angiogenic stimulation of MSC^90^. However, as direct evidence supporting the involvement of these adipokines in the dysfunction of obese MSC is lacking, further studies will be required to gain better insights into the modulation of MSC fate by these adipokines during obesity.

**Fig. 3:**
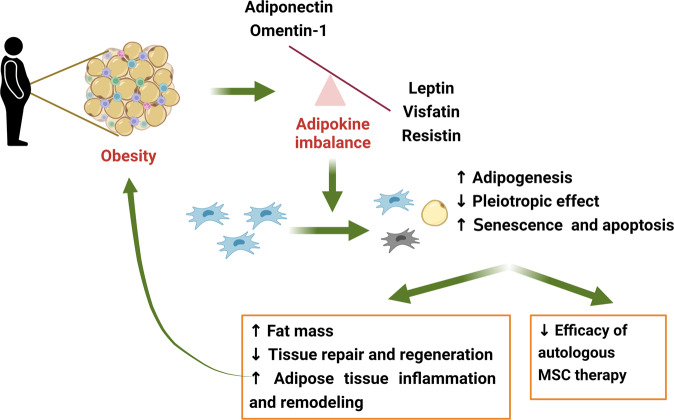
Putative roles of adipokines in MSC dysfunction during obesity. Obesity induces an imbalance in the production and secretion of adipokines. Some adipokines, such adiponectin and omentin-1, are downregulated, whereas most of others are upregulated in response to excess adiposity. High levels of leptin, visfatin, and resistin in the MSC niche promote the differentiation of MSC to adipocytes, impaired pleiotropic functions, and accelerate senescence and apoptotic cell death. In addition, downregulation of adiponectin and omentin-1, which play a critical role in the maintenance of survival and functional plasticity of MSC, further contributes to impaired MSC functions. Consequently, obese-derived MSC has exhibited the lower efficacy in treatment of various disorders. Given the role of MSC as critical regulators of inflammatory and immune responses, adipogenesis, and tissue repair and regeneration, abnormal function of MSC in obese subjects would exacerbate adipose tissue (AT) inflammation and remodeling, as well as promote the initiation and progression of obesity-associated complications.

In summary, although the effects of adipokines on MSC fate are not always consistent across different studies, anti-inflammatory adipokines, including adiponectin, apelin, and omentin-1, appear to exert beneficial effects on the maintenance of stemness, differentiation potential, functional plasticity, and survival of MSC under stressful conditions. In contrast, long-term exposure to high concentrations of pro-inflammatory adipokines, including leptin, NAMPT/Visfatin, and resistin, may promote cellular senescence and apoptosis, as well as alter differentiation pattern of MSC. Given the fact that protective adipokines (i.e., adiponectin and omentin-1) are downregulated, but pro-inflammatory adipokines are increasingly produced during obesity, the abnormal behaviors of obese-derived MSC may be attributed to the imbalance in adipokine production. Since functional MSC are required to control adipogenesis/osteogenesis balance, regulate inflammatory and immune responses, and mediate tissue repair and regeneration, obesity-induced MSC dysfunction further fosters the progression of obesity-linked metabolic syndromes. The putative roles of adipokines in the crosstalk between obesity and MSC fate are illustrated in Fig. 3.

## MSC therapy: a promising strategy for normalization of adipokine profile and counteraction of obesity

**Fig. 4:**
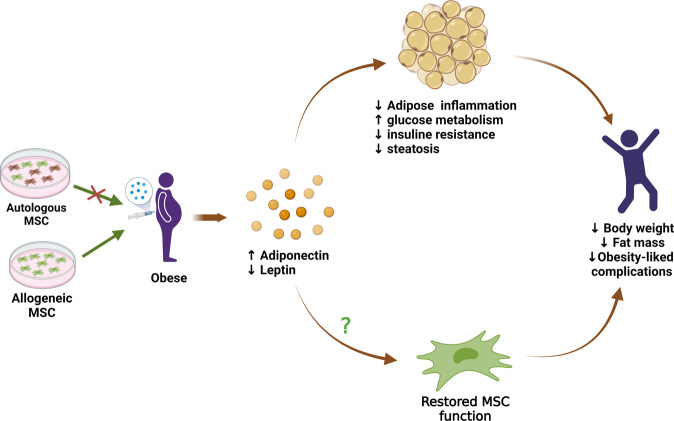
Roles of adipokine rebalancing in MSC therapy for the treatment of obesity. MSC therapy has been emerging as a promising strategy for counteracting obesity and its related complications. Autologous and allogenic stem cells can be used for MSC therapy; however, obese-derived autologous MSC may be therapeutically ineffective due to compromised potency. Among various benefits, MSC treatment increases adiponectin and reduces leptin levels, thereby re-establishing the adipokine balance in subjects with obesity. Since adipokines exert a variety of effects on inflammation, metabolism, and energy expenditure, normalization of serum adipokines is hypothesized to contribute to other therapeutic benefits of MSC, including improvements in the metabolic profiles of glucose and lipids decrease in adipogenesis, amelioration of adipose tissue inflammation and remodeling, and prevention of obesity-induced metabolic complications. Furthermore, restoration of the adipokine profile may retrain MSC and restore their normal functions, which in turn can reverse the progression of obesity.

As previously discussed, endocrine and metabolic alterations during obesity trigger MSC dysfunction, as corroborated by certain MSC properties, such as restricted plasticity, impaired pleiotropic activity, and increased sensitivity to senescence and apoptotic cell death. Since MSC are required for the repair and regeneration of injured tissues, the development of obesity-associated complications may be ascribed, at least in part, to the compromised protective function of these cells. Hence, it would stand to reason that administration of functional MSC to obese patients may prevent or reverse the progression of obesity-related comorbidities. Despite the lack of direct clinical evidence, a large amount of preclinical data have revealed the effectiveness of MSC therapy. In most studies, treatment of diet-induced obese (DIO) mice with human MSC or MSC-derived conditioned media led to decreased body weight and fat mass^91^. More importantly, MSC therapy ameliorated the metabolic profile in obese mice by increasing insulin sensitivity and decreasing blood glucose and triglyceride levels^91^. As a result, repeated administration of MSC protected obese mice from the development of obesity-associated metabolic syndromes, such as diabetes, fatty liver disease, and cardiovascular dysfunctions^92,93^. Intriguingly, MSC therapy could alleviate adipose tissue inflammation and rebalance serum adipokine levels. Indeed, injection of conditioned media from tonsil‑derived MSC was reported to increase the expression of adiponectin in epididymal adipose tissue, as well as circulating levels of total and high-molecular-weight adiponectin in obese mice^94^. Similarly, treatment with BM-MSC or their conditioned media increased adiponectin gene expression in HFD-fed mice, which was accompanied by protection against obesity-induced cardiac dysfunction^95^. In addition, human adipose-derived MSC-based therapy alleviated serum leptin levels and/or the leptin-adiponectin ratio, which is an important index for predicting the risk of obesity-related disorders^96,97^. Taking into consideration the critical role of adipokines in the regulation of MSC fate, normalization of adipokine profile by MSC therapy might be helpful in counteracting obesity-induced MSC dysfunction, highlighting a bidirectional relationship between adipokines and MSC during obesity (Fig. 4). Nevertheless, it remains to be verified whether MSC therapy and adipokine rebalancing can restore the functional properties of MSC in individuals with obesity.

A limitation of the current understanding of MSC-based therapy is that most, if not all, studies have used lean MSC to treat obese subjects. While obese-derived MSC exhibits clearly compromised potency, direct evidence regarding their effectiveness in the treatment of obesity is still lacking. Although MSC are believed to possess low immunogenicity that protects them from immune rejection and supports the use of allogeneic MSC, the heterogeneous nature of MSC makes them a risk for stimulating the immune response, especially the rate of allograft rejection is found to be higher in obese subjects^98^. Therefore, it would still be of interest to develop, if possible, strategies to retrieve the function of autologous MSC in patients with obesity. On the other hand, the recipient’s microenvironment has been postulated to have a significant impact on the effectiveness of MSC therapy. For example, animal studies using models of graft-versus-host disease (GVHD) have shown that MSC efficacy was largely dependent on serum levels of pro-inflammatory cytokines and the abundance of peripheral blood lymphocytes^99^. Unfortunately, although obesity is known to induce significant changes in both local and systemic microenvironments, its impact on the behaviors of transplanted MSC is currently under-investigated. One of the critical factors under consideration would be the aberrant adipokine profile in obese individuals. In fact, in addition to the differential modulation of MSC fate by adipokines presented in previous sections, there has been evidence that impaired adiponectin signaling and/or elevated leptin levels increase the risk of allograft rejection^100^. Therefore, normalization of adipokine signaling might be helpful in optimizing the effectiveness of MSC therapy for obese patients.

## Conclusions and perspectives

In conclusion, adipokines have emerged as key determinants of MSC fate in the local and systemic milieus of patients with obesity. Dysregulation of adipokine secretion, which is characterized by decreases in the levels of adiponectin and increases in the levels of leptin, NAMPT/Visfatin, and/or resistin, triggers abnormalities in behaviors of both resident and transplanted allogeneic MSC, which in turn further promote obesity-associated disorders and alter the way obese patients respond to MSC therapy. While MSC have demonstrated their great potential to serve as a promising treatment strategy for obesity and comorbidities, MSC therapy is still far from optimal. The optimization of MSC therapy will require further insights into how obese environment affects the functional attributes of MSC. In this scenario, it would be important to determine whether obesity-induced alterations in MSC functionality are reversible. With regard to the central role of adipokines in the interactions between MSCs and the obese environment, targeting adipokine signaling in obese patients or retraining obese MSC with certain adipokines might be potential approaches to counteract the detrimental effects of obesity on MSC functionality and improve the effectiveness of MSC therapy.
